# A Cost-Effective Geodetic Strainmeter Based on Dual Coaxial Cable Bragg Gratings

**DOI:** 10.3390/s17040842

**Published:** 2017-04-12

**Authors:** Jihua Fu, Xu Wang, Tao Wei, Meng Wei, Yang Shen

**Affiliations:** 1 Key Laboratory of Crustal Dynamics, Institute of Crustal Dynamics, China Earthquake Administration; Beijing 100085, China; wangxu19920601@126.com; 2 Engineering Department, University of Rhode Island, Kingston, RI 02881, USA; wei@ele.uri.edu; 3 Graduate School of Oceanography, University of Rhode Island, Narragansett, RI 02882, USA; matt-wei@uri.edu (M.W.); yshen@gso.uri.edu (Y.S.)

**Keywords:** surface deformation, geodetic strainmeter, Bragg grating, coaxial cable

## Abstract

Observations of surface deformation are essential for understanding a wide range of geophysical problems, including earthquakes, volcanoes, landslides, and glaciers. Current geodetic technologies, such as global positioning system (GPS), interferometric synthetic aperture radar (InSAR), borehole and laser strainmeters, are costly and limited in their temporal or spatial resolutions. Here we present a new type of strainmeters based on the coaxial cable Bragg grating (CCBG) sensing technology that provides cost-effective strain measurements. Two CCBGs are introduced into the geodetic strainmeter: one serves as a sensor to measure the strain applied on it, and the other acts as a reference to detect environmental noises. By integrating the sensor and reference signals in a mixer, the environmental noises are minimized and a lower mixed frequency is obtained. The lower mixed frequency allows for measurements to be taken with a portable spectrum analyzer, rather than an expensive spectrum analyzer or a vector network analyzer (VNA). Analysis of laboratory experiments shows that the strain can be measured by the CCBG sensor, and the portable spectrum analyzer can make measurements with the accuracy similar to the expensive spectrum analyzer, whose relative error to the spectrum analyzer R3272 is less than ±0.4%. The outputs of the geodetic strainmeter show a linear relationship with the strains that the CCBG sensor experienced. The measured sensitivity of the geodetic strainmeter is about −0.082 kHz/με; it can cover a large dynamic measuring range up to 2%, and its nonlinear errors can be less than 5.3%.

## 1. Introduction

Observations of surface deformation are essential for understanding a wide range of geophysical problems, including earthquakes, volcanoes, landslides, and glaciers. More than 1200 Global Positioning System (GPS) stations onshore and five seafloor geodetic stations offshore were installed in Japan [1,2]. Additionally, in America, the Plate Boundary Observatory (PBO) plan was founded by the National Science Foundation (NSF) as the geodetic component of the EarthScope project. The PBO consists of a network of 1100 permanent, continuously operating GPS stations, 74 borehole strainmeters, 26 shallow borehole tiltmeters, and six long baseline laser strainmeters [3]. Since 2012, the Crustal Movement Observation Network of China (CMONoC) was built. Included in the CMONoC are 260 continuous observation stations and 2000 irregular observation stations [4]. The geodetic technologies used in these three networks, such as GPS, InSAR, borehole and laser strainmeters, provide critical observations of surface deformation and reveal the detailed processes of seismogenic faults. However, the current geodetic technologies are costly and limited in their temporal or spatial resolutions [5,6,7]. Cost-effective and distributed strainmeters are desired for geodic applications. Fiber optic sensors, such as fiber Bragg grating (FBG), represent an opportunity for distributed strain sensing [8]. However, limited by its silica glass nature, the fiber optic sensor will easily break when it is subjected to large strains (about 10 mε or 1%) and/or a shear force [9]. Inspired by the FBG sensor, a new coaxial cable Bragg grating (CCBG) sensor was put forward, which is sufficiently robust to survive and operate on large strains, while it holds comparable performances with the FBG sensor, such as distributed sensing, cost-effectiveness, and accuracy [10,11]. A cost-effective geodetic strainmeter was designed with dual CCBGs to obtain a lower mixed frequency and minimize environmental noises.

## 2. Working Principle of CCBG

**Figure 1:**
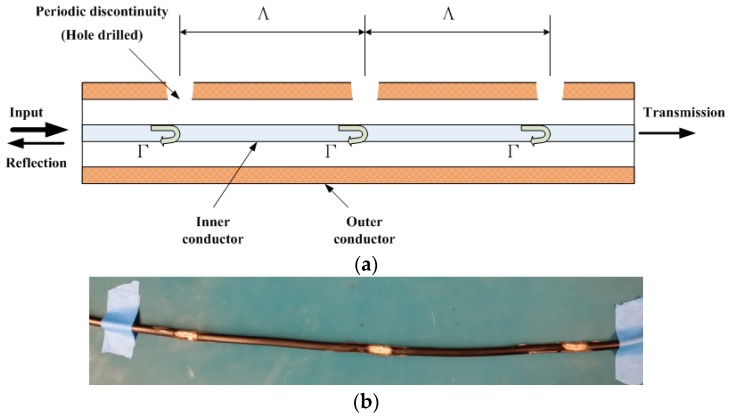
The structure of the coaxial cable Bragg grating (CCBG) sensor: (**a**) Depiction of the CCBG sensor’s working principle; (**b**) Depiction of the CCBG sensor’s real appearance.

Following the similar fundamental electromagnetic (EM) theory with the FBG, the CCBG is an elastic coaxial cable with some periodic impedance discontinuities in the dielectric insulator and outer conductor along the length of the cable, an illustration of which is shown in Figure 1. When Radio Frequency (RF) signals are loaded into the CCBG, some of the RF signals will be reflected on each of these discontinuities and some of the RF signals will be transmitted. The reflected signal, or resonance signal, is enhanced by the numbers of these discontinuities. Similar to the well-studied case of fiber Bragg gratings, the forward propagating mode is coupled to the backward propagating mode in the waveguide at discrete resonant frequencies, satisfying the following Bragg condition [8].

(1) $$\beta^{+}-\beta^{-}=2\beta =\frac{2m\pi }{\Lambda }\;\mathrm{or}\;f_{res}^{m}=\frac{m}{2\Lambda \sqrt{\mathrm{LC}}},$$

where *β*^+^ and *β*^−^ are the propagation constants of the forward and backward traveling waves, respectively. They have the same magnitude, but opposite signs. Λ is the distance of the periodic discontinuities. L and C represent the inductance and capacitance of the cable, respectively. The resonant frequency is represented as *f*_res_, and *m* is an integer representing the diffraction order of the grating.

When a force is applied to the CCBG, a resonant frequency shift is introduced because of the change of the cable dimension and dielectric constant of the inner dielectric material. The strain-induced resonant frequency shift can be expressed by the following [12]:

(2) $$\Delta f_{res}=-\frac{L_{s}}{L_{t}}\cdot \left[\frac{\Delta L}{L_{s}}-\frac{\Delta \sqrt{\varepsilon_{r}}}{\sqrt{\varepsilon_{r}}}\right]\cdot f_{res}=-\frac{L_{s}}{L_{t}}\cdot (1-P_{eff})\cdot \varepsilon \cdot f_{res},$$

where *L_s_* is the sensing length of CCBG, *L_t_* is the total length of the cable under test, Δ*L* is the change in length of CCBG, $\Delta \sqrt{\varepsilon_{r}}$ is the change in square root of relative permittivity of the dielectric material, $\sqrt{\varepsilon_{r}}$ is the square root of relative permittivity of the dielectric material, *P_eff_* is the effective coefficient, and *ɛ* is the strain applied. Through simplifying and calculating (*L_s_* = *L_t_*, and *P_eff_* = 0.2216), the sensitivity of CCBG can be obtained by the equation below:

(3) $$\frac{\Delta f}{\mu \varepsilon }=-0.7784\times 10^{-6}f_{res}(\mathrm{Hz}/\mu \varepsilon ),$$

Using Equation (2) to obtain the strain applied, the resonant frequency shift should be obtained first. Also, the resonant frequency shift can be measured by a vector network analyzer (VNA). Although CCBGs can be joined together to build a distributed network, the VNA is considered too expensive for many applications. To solve this problem, a positive feedback system is introduced to enhance the sensitivity of the CCBG sensor [13]. The sensor’s Q factor is improved, making it possible to measure the strain applied on the CCBG sensor with a cheap spectrum analyzer (SA), rather than an expensive VNA.

**Figure 2:**
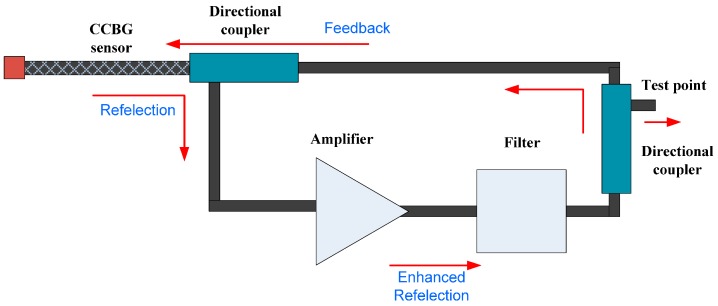
The system diagram of the positive feedback system.

As shown in Figure 2, the positive feedback system is composed of a CCBG sensor, an amplifier, a group of filters and two directional couplers, which is developed based on the idea of an oscillator. The reflection (resonant frequency signal) of the CCBG is directed to an amplifier by one of the directional couplers. Then, the reflection will be amplified by the amplifier and be selected by a given filter. Through the other directional coupler, the enhanced reflection will be fed back to the CCBG. The reflection or resonant frequency signal is constantly strengthened in this positive feedback loop. On the second directional coupler there is a test point, which is used to test the reflection’s frequency. If the test point is connected to a spectrum analyzer, then the resonant frequency can be measured. Furthermore, the resonant frequency shift represents the strain applied on the CCBG. The positive feedback system enhances the CCBG sensor sensitivity, in addition to reducing the cost of sensing.

## 3. Geodetic Strainmeter Design

### 3.1. Hardware Design

A spectrum analyzer is cheaper than a VNA, but as a lab instrument it is also big and expensive. Consequently, for a cost-effective geodetic application, cheap and portable equipment is expected. Although the spectrum analyzer cannot be used directly, its working principles can work well. Based on the Heterodyne principle, a geodetic strainmeter is designed.

**Figure 3:**
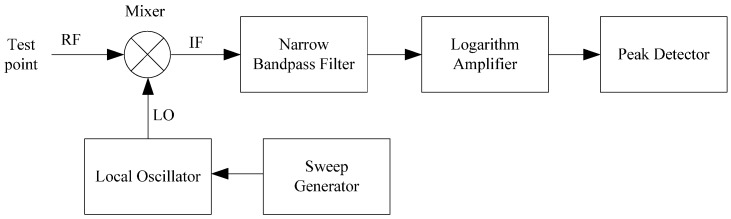
The block diagram of a spectrum analyzer based on the Heterodyne principle.

As shown in Figure 3, the spectrum analyzer consists of a sweep generator, local oscillator, mixer, narrow bandpass filter, logarithm amplifier and peak detector. The signal (radio-frequency, RF) from the test point of the CCBG sensor is mixed with the output of a local oscillator (LO) in the mixer. The mixer’s output is an intermediate-frequency (IF) signal, and its frequency can be described by the following:

(4) $$f_{IF}=\pm mf_{LO}\pm nf_{RF},$$

where *f_IF_* is the frequency of the IF signal, *f_LO_* is the frequency of the LO signal, *f_RF_* is the frequency of the RF signal, and *m* and *n* are the integral orders of the LO and RF signals. Usually, the first orders of the LO and RF signals are used in the real application system. Moreover, Equation (4) is often simplified by:

(5) $$f_{IF}=f_{LO}-f_{RF},$$

Then the RF signal is sent to the narrow bandpass filter for frequency selecting. After that, the filtered signal is sent to the logarithm amplifier. Through the logarithm amplifier the IF signal’s intensity in the narrow passband is converted into power. When the local oscillator is tuned by the sweep generator, by Equation (4) or Equation (5), the IF signal is also changed in the same way as the LO signal. The filter’s passband is narrow and fixed. In the frequency domain, the tuned IF signal passes through the passband of the narrow bandpass filter sequentially, which is illustrated by the sketch below.

**Figure 4:**
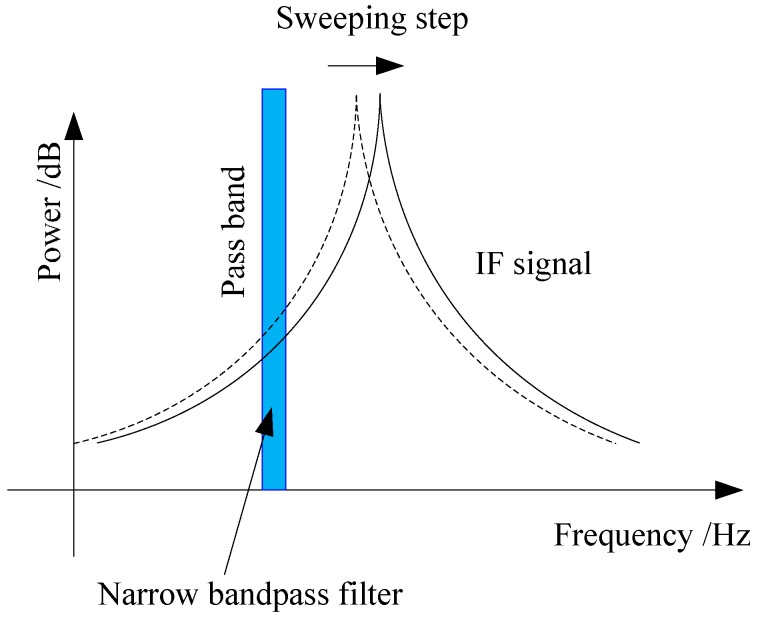
The diagrammatic sketch for spectrum analyzing based on the Heterodyne principle.

Shown by Figure 4, the full spectrum of the IF and RF signals can be figured out step by step by the sweeping process. Then, with the help of the peak detector, the maximum of the spectrum can be obtained. The frequency corresponding to the maximum spectrum represents the resonant frequency of the CCBG sensor.

**Figure 5:**
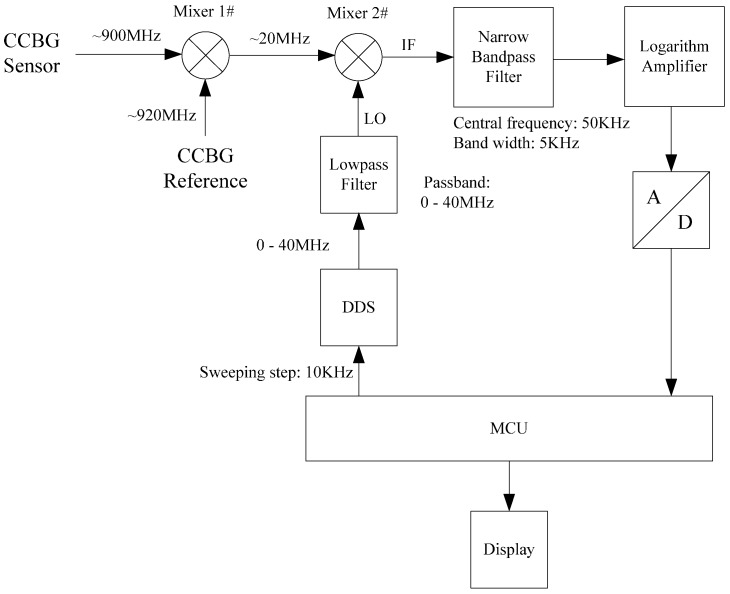
The block diagram of the geodetic strainmeter with dual CCBGs.

Since the resonant frequency is high (usually higher than 900 MHz), the subsequently required high frequency, high precision and tunable local oscillator is complex and expensive. To cut down the cost of the geodetic strainmeter, the frequency on the test point should be decreased first. Taking this idea, another mixer and a reference CCBG are both introduced into the strainmeter design. Its working principle is shown in Figure 5.

Shown by Figure 5, two CCBGs are used in the geodetic strainmeter: one is used to measure the strain applied on it, and the other acts as a reference only to detect environmental noises. For example, the CCBG sensor’s resonant frequency is set to about 900 MHz, and the CCBG reference’s resonant frequency is set to about 920 MHz. Through Mixer 1, the signal’s frequency can be cut down to about 20 MHz. Thus, the output of Mixer 1 becomes low enough to be measured by a cheap and portable spectrum analyzer. The portable spectrum analyzer is a microcontroller unit (MCU) based system. There, a Direct Digital Synthesizer (DDS) and a low pass filter serve as the local oscillator in Figure 3. In addition, the MCU acts as the sweep generator. Its sweeping range is from 10 MHz to 30 MHz, and its sweeping step is set to 10 KHz. The narrow bandpass filter’s central frequency is 50 KHz and its band width is 5 KHz. The output of the narrow bandpass filter is sent to a logarithm amplifier to calculate its power. Then the logarithm amplifier’s output is sampled by an Analog-to-Digital (A/D) convertor. Under the control of the MCU, the signal’s power in the passband is converted into digital signal and stored in the MCU. Also, the MCU also works as a peak detector to figure out the resonant frequency.

### 3.2. Software Design

**Figure 6:**
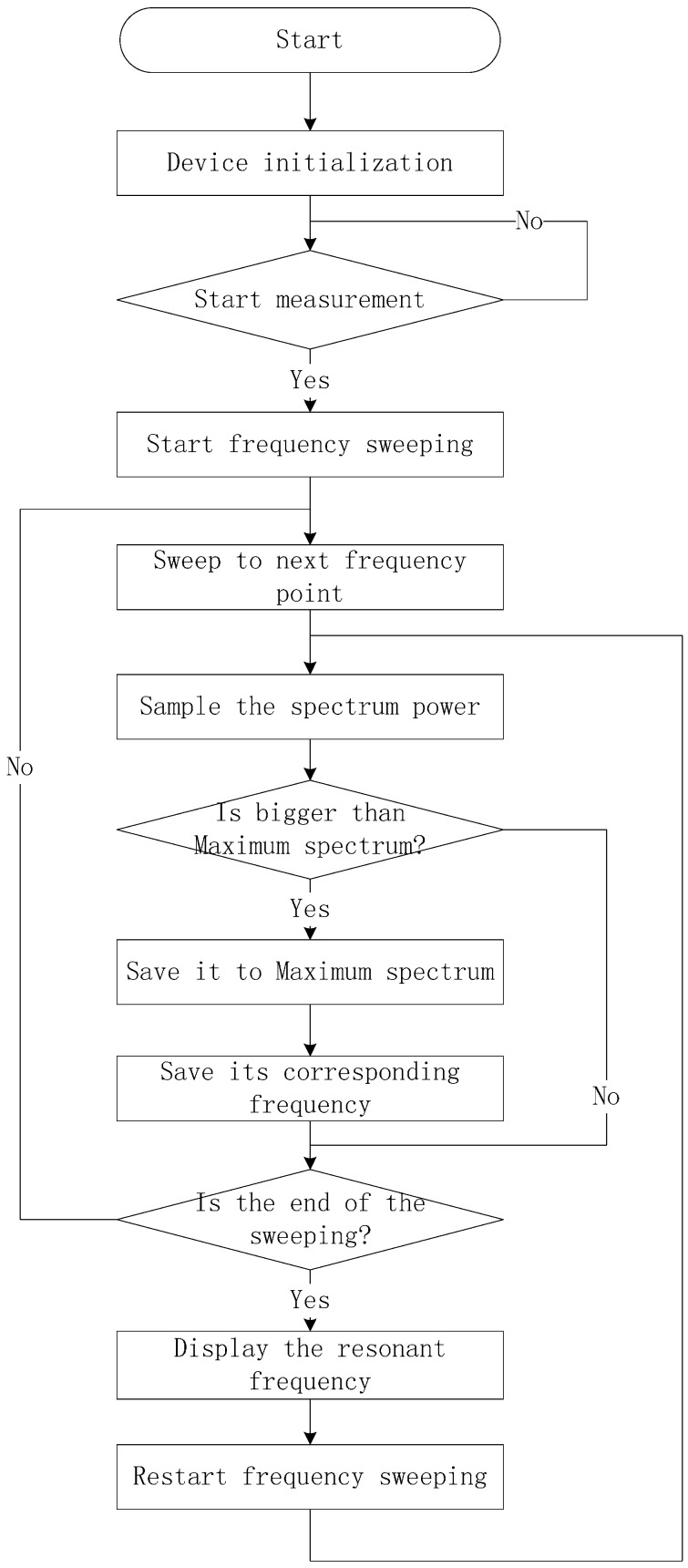
The flow chart of the geodetic strainmeter.

The software of the geodetic strainmeter is realized by the programs in the MCU. The programs tune the DDS to generate the sweeping LO signal, control the A/D convertor to sample the filtered IF signal, and build an inside peak detector to catch the maximum spectrum and its corresponding resonant frequency of the CCBG sensor. The flow chart of the MCU is shown in Figure 6.

When the geodetic strainmeter is opened, the device will first be initialized. On this condition, the MCU will do nothing until the start measurement command is received. Then, the MCU will start the frequency sweeping. The MCU sweeps the LO to a next frequency point, and samples the spectrum power of the filtered IF signal. If the spectrum power is bigger than the maximum spectrum stored in MCU, then the spectrum power and its frequency will be saved to the maximum spectrum and its corresponding frequency. If the end of the sweeping arrives, the resonant frequency will be displayed on the liquid crystal display (LCD) and the MCU will restart a new frequency sweeping. Otherwise, the MCU will sweep the LO to the next frequency point and do the loop above, until the sweeping comes to an end.

## 4. Demo System

**Figure 7:**
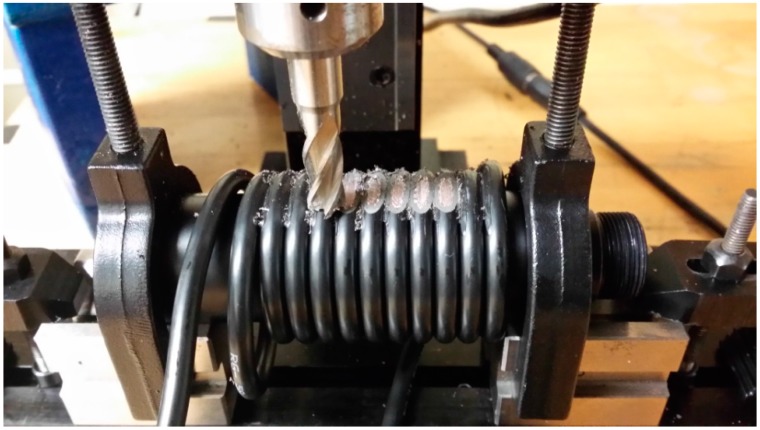
The CCBG produced on a milling machine.

A demo system was built according to the block diagram shown by Figure 5. As shown in Figure 7, the CCBG sensor and the CCBG reference were both built of coaxial cable (50 Ω, Jamco Electronics, RG-58, Belmont, CA, USA) by a precision milling machine.

**Figure 8:**
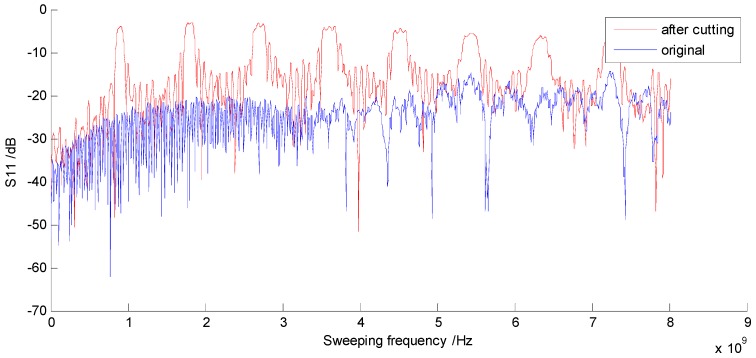
These are S11 data collected before and after cutting: the blue line is the original data before cutting; the red line is the new data after cutting.

The coaxial cable was wound around a rod first, and then the dielectric insulator and outer conductor were cut out by the milling machine at the same time. By this method, the amount of cutting was well maintained. The distance of the periodic discontinuities Λ of the CCBG sensor is about 11.2 cm. According to the cable parameters from datasheet, its distributed capacitance C is 100 pF/m and its distributed inductance L is 250 nH/m. By Equation (1), the first order of the resonant frequencies is about 892.9 MHz. Before and after cutting, the reflection parameter S11 of the coaxial cable was tested by a VNA (Agilent, Santa Clara, CA, USA, N3383A), and the data collected are shown in Figure 8. The first order of the resonant frequencies is 907.4 MHz in reality, which is close to the theory value and supports the Bragg condition.

**Figure 9:**
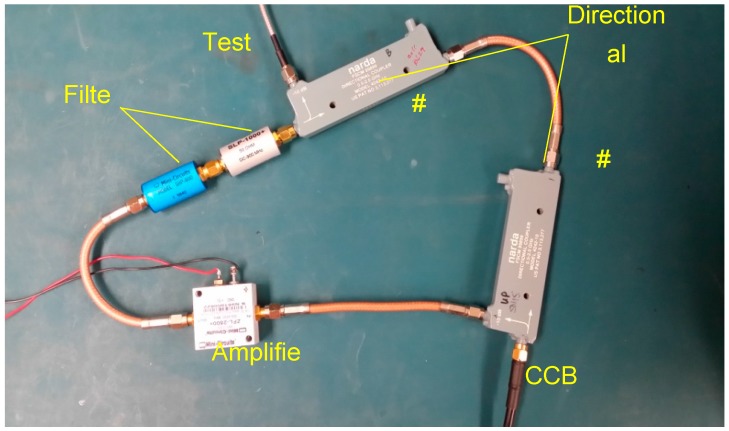
The demo system of CCBG’s positive feedback system.

To enhance the CCBG’s sensitivity, the positive feedback system was built, which is shown in Figure 9. There, the gain of the amplifier is up to 30 dB, and the passband of the filters is from 800 MHz to 1.2 GHz.

The portable spectrum analyzer was designed with TI’s MCU MSP430F169. The spectrum analyzer’s parameters were selected according to the real application needs, which are shown below in Table 1.

The demo system’s measuring range *D* and *R* resolution are respected as ±20,000 με (2%) and 20 με, respectively. The resonant frequency of the CCBG sensor *f*_res_ is selected to be 900 MHz. According to Equation (3), the sensitivity of the CCBG sensor is −0.7 kHz/με. Consequently, the CCBG sensor’s resolution in frequency *R_f_* is 14.00 kHz, and the CCBG sensor’s output range is 900 ± 14 MHz. The resonant frequency of the CCBG reference *f*_re__f_ is selected to be 920 MHz. The narrow bandpass filter’s central frequency *f*_0_ is set to 50 kHz and its band width *f*_w_ is set to 5 kHz. The band width *f*_w_ should be less than half of the resolution in frequency. The sweeping frequency range of the DDS is from 0 to 40 MHz, which should be greater than 20 ± 14 MHz. Also, the sweeping step of the DDS is set to 10 kHz, which should be less than the resolution in frequency *R_f_*. The low pass filter’s passband is from 0 to 40 MHz, which is the same as the DDS’s sweeping frequency.

## 5. Results

**Figure 10:**
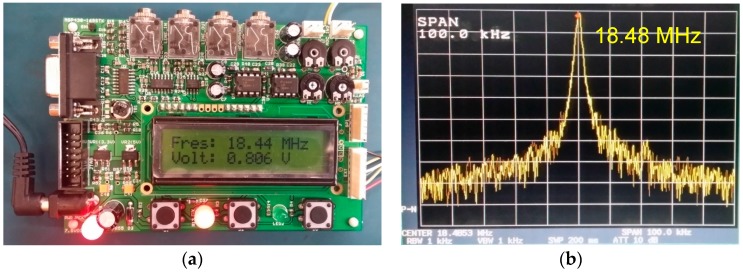
The test results of the portable spectrum analyzer and the spectrum analyzer R3272: (**a**) The result obtained by the portable spectrum analyzer; (**b**) The result obtained by the spectrum analyzer R3272.

To evaluate the performance of the demo system, the MCU-based portable spectrum analyzer’s measurement results were compared with those of a spectrum analyzer (ADVANTEST R3272, Baldwin, CA, USA). Stresses were applied onto the CCBG sensor, while the CCBG reference was left unrestrained (free of stress). The output of Mixer 1 in Figure 5 was tested by our portable spectrum analyzer and the spectrum analyzer R3272 at the same time. The test results are shown in Figure 10.

Shown in Figure 10, the peak frequency of 18.44 MHz obtained by the portable spectrum analyzer is close to the 18.48 MHz obtained by the spectrum analyzer R3272. Similar results were also achieved when some other stresses were applied, which are shown in Table 2. According to the test results, the portable spectrum analyzer could measure the IF signal’s peek frequency with high accuracy, with relative errors to the spectrum analyzer R3272 less than ±0.4%.

**Figure 11:**
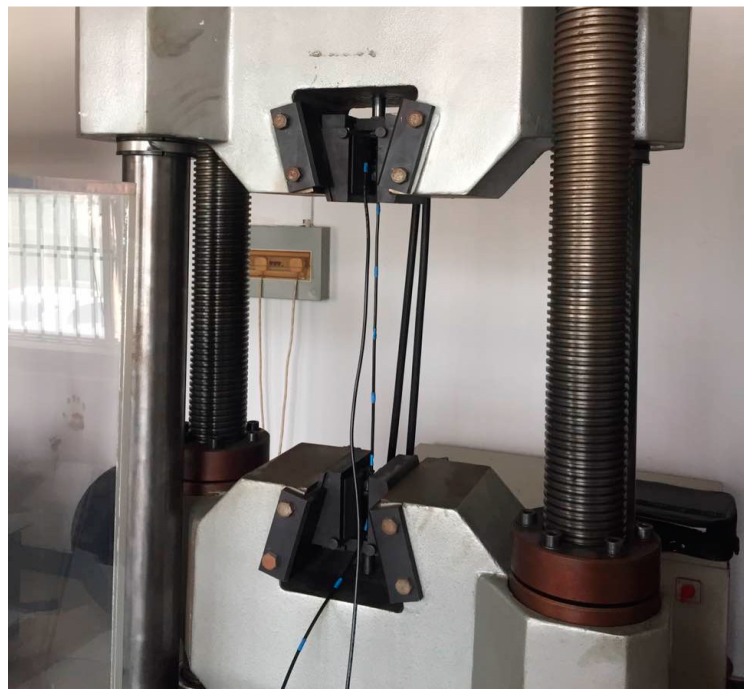
The experimental setup for the axially tensile strain test.

For axially tensile strain test, the CCBG sensor was mounted onto a universal material testing machine (WAP-1000, Zhongluchang Co., Jinan, China), as shown in Figure 11.

**Figure 12:**
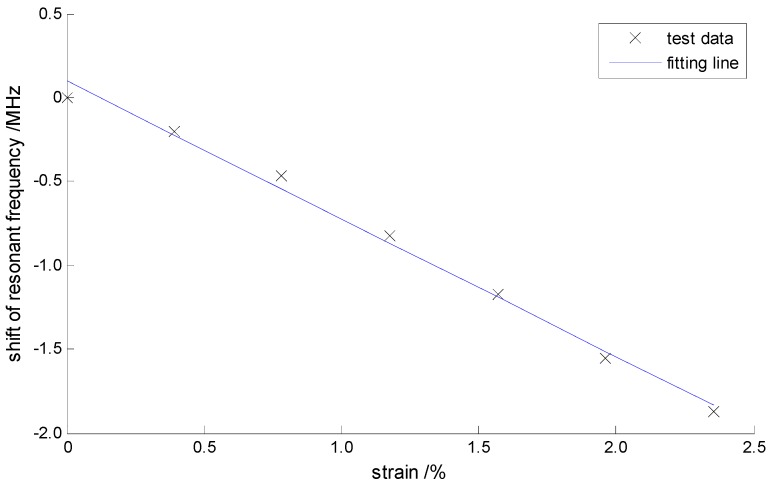
The test results of the axially tensile strain.

One end of the CCBG sensor was mounted on the fixed frame (the upper one) of the WAP-100, and the other end was attached to the moveable frame (the lower one) of the WAP-100. The movement of the lower frame was accurately controlled to move certain displacements. And the deformation along the CCBG sensor is equal to the displacement of the lower frame. The displacements of the lower frame were set to change from 0 to 12 mm, with steps of 2 mm. Because the length of the CCBG sensor under strain is 51 cm, the strain variation per step is 0.39% (3900 με). At all steps the shifts of the resonant frequency were obtained, which are shown in Table 3 and Figure 12.

The shifts of the resonant frequency show a linear relationship with the strains that the CCBG sensorexperienced. A least squares fitting line was added in Figure 12, with which the nonlinear errors were calculated and shown in the Table 3. The axially tensile strain test shows that the CCBG sensor can measure the strain greater than 2% (>2.35%), and its nonlinear errors can be less than 5.3%. The measured sensitivity of the geodetic strainmeter is about −0.082 kHz/με and it is less than the designed sensitivity (−0.7 kHz/με). This could possibly be caused by the conversion of the resonant frequency from a high radio frequency to a lower intermediate-frequency, described by Equation (4).

## 6. Conclusions

Two CCBGs are introduced into the geodetic strainmeter: one serves as a sensor to measure the strain applied on it, and the other acts as a reference to detect environmental noises. Their output signals are mixed together in a mixer. By this method, the environmental noises can be minimized and, more importantly, the signal’s frequency to be processed can be reduced to a lower level. The lower frequency is preferable because it can be easily processed by a portable spectrum analyzer rather than an expensive spectrum analyzer or VNA. Therefore, the strain sensing cost can be cut down significantly. Based on this idea, a demo system of the cost-effective geodetic strainmeter was built. Analysis of laboratory experiments shows that the strain can be measured accurately by a CCBG sensor. Additionally, the portable spectrum analyzer’s relative errors are shown to be less than ±0.4%, compared to the spectrum analyzer R3272. This indicates that the portable spectrum analyzer measures to a similar accuracy as the spectrum analyzer. Through axially tensile strain test, it is shown that the outputs of the geodetic strainmeter show a linear relationship with the strains that the CCBG sensor experiened. Although the measured sensitivity of the geodetic strainmeter is about −0.082 kHz/με, less than the designed sensitivity, it can cover a large dynamic range up to 2%, and its nonlinear errors can be less than 5.3%. Further research on how the sensitivity of the strainmeter can be improved will be the main work of the next study. Moreover, some field tests and distributed tests will be performed in the near future.

## Figures and Tables

**Table 1:** The demo system’s parameters selected.

Part	Item	Symbol	Value	Comments
Demo system	Measuring range	*D*	±20,000 με (2%)	respected
	Resolution	*R*	20 με	respected
CCBG sensor	Resonant frequency	*f*_res_	900 MHz	designed
	Theory sensitivity	*A*	−0.7 kHz/με	by Equation (3)
	Resolution in frequency	*R_f_*	14.00 kHz	*|AR|*
	Output Range		900 ± 14 MHz	*f*_res_ ± *|AD|*
CCBG reference	Resonant frequency	*f*_ref_	920 MHz	designed
Narrow bandpass filter	Central frequency	*f*_0_	50 kHz	designed
	Band width	*f*_bw_	5 kHz	<*R_f_*/2
DDS	Sweeping frequency	*f*_wp_	0 to 40 MHz	>(*f*_ref_ − *f*_res_) ± *|AD|*
	Sweeping step	*f*_stp_	10 kHz	<*R_f_*
Low pass filter	Passband		0 to 40 MHz	same as *f*_wp_

**Table 2:** The results comparison between the portable spectrum analyzer and the R3272.

No.	Portable Spectrum Analyzer’s/MHz	R3272’s/MHz	Relative Error/%
1	18.44	18.48	−0.22
2	17.32	17.38	−0.34
3	16.56	16.59	−0.18
4	15.87	15.92	−0.31

**Table 3:** The test results of the axially tensile strain.

No.	Strains/%	Frequency Shifts/MHz	Nonlinear Errors/%
1	0	0	5.28
2	0.392%	−0.20	−1.36
3	0.784%	−0.47	−4.19
4	1.176%	−0.82	−2.65
5	1.569%	−1.17	−1.11
6	1.961%	−1.55	2.07
7	2.353%	−1.87	1.97
